# Oral fexinidazole for stage 1 or early stage 2 African *Trypanosoma brucei gambiense* trypanosomiasis: a prospective, multicentre, open-label, cohort study

**DOI:** 10.1016/S2214-109X(21)00208-4

**Published:** 2021-06-15

**Authors:** Victor Kande Betu Ku Mesu, Wilfried Mutombo Kalonji, Clélia Bardonneau, Olaf Valverde Mordt, Digas Ngolo Tete, Séverine Blesson, François Simon, Sophie Delhomme, Sonja Bernhard, Hélène Mahenzi Mbembo, Christian Mpia Moke, Steven Lumeya Vuvu, Junior Mudji E'kitiak, Felix Akwaso Masa, Melchias Mukendi Ilunga, Dieudonné Mpoyi Muamba Nzambi, Tim Mayala Malu, Serge Kapongo Tshilumbwa, Franck Botalema Bolengi, Mathieu Nkieri Matsho, Crispin Lumbala, Bruno Scherrer, Nathalie Strub-Wourgaft, Antoine Tarral

**Affiliations:** a Ministry of Health, Kinshasa, Democratic Republic of the Congo; b National HAT Control Programme, Programme National de Lutte contre la Trypanosomiase Humaine Africaine, Kinshasa, Democratic Republic of the Congo; c Drugs for Neglected Diseases Initiative, Geneva, Switzerland; d Drugs for Neglected Diseases initiative, Kinshasa, Democratic Republic of the Congo; e Swiss Tropical and Public Health Institute, Basel, Switzerland; f Bandundu Hospital, Kwilu Province, Democratic Republic of the Congo; g Vanga Hospital, Kwilu Province, Democratic Republic of the Congo; h Masi Manimba Hospital, Kwilu Province, Democratic Republic of the Congo; i Dipumba Hospital (MIBA), Mbuji Mayi, Kasaï Oriental Province, Democratic Republic of the Congo; j Mushie Hospital, Maï Ndombe Province, Democratic Republic of the Congo; k Katanda Hospital, Kasaï Oriental Province, Democratic Republic of the Congo; l Isangi Hospital, Tshopo Province, Democratic Republic of the Congo; m Bagata Hospital, Kwilu Province, Democratic Republic of the Congo; n Bruno Scherrer Conseil, Saint Arnoult en Yvelines, France

## Abstract

**Background:**

Staging and treatment of human African trypanosomiasis caused by *Trypanosoma brucei gambiense* (g-HAT) required lumbar puncture to assess cerebrospinal fluid (CSF) and intravenous drugs that cross the blood–brain barrier for late-stage infection. These procedures are inconvenient in rural health systems of disease-endemic countries. A pivotal study established fexinidazole as the first oral monotherapy to be effective against non-severe stage 2 g-HAT. We aimed to assess the safety and efficacy of fexinidazole in early g-HAT.

**Methods:**

In this prospective, multicentre, open-label, single-arm cohort study, patients with stage 1 or early stage 2 g-HAT were recruited from eight treatment centres in the Democratic Republic of the Congo. Primary inclusion criteria included being older than 15 years, being able to ingest at least one complete meal per day (or at least one sachet of Plumpy'Nut®), a Karnofsky score higher than 50, evidence of trypanosomes in the blood or lymph but no evidence of trypanosomes in the CSF, willingness to be admitted to hospital to receive treatment, having a permanent address, and being able to comply with the follow-up visit schedule. Exclusion criteria included severe malnutrition, inability to take medication orally, pregnant or breastfeeding women, any clinically important medical condition that could jeopardise patient safety or participation in the study, severely deteriorated general status, any contraindication to imidazole drugs, HAT treatment in the past 2 years, previous enrolment in the study or previous intake of fexinidazole, abnormalities on electrocardiogram that did not return to normal in pretreatment repeated assessments or were considered clinically important, QT interval corrected using Fridericia's formula of at least 450 ms, and patients not tested for malaria or not having received appropriate treatment for malaria or for soil-transmitted helminthiasis. Patients were classified into stage 1 or early stage 2 g-HAT groups following evidence of trypanosomes in the blood, lymph, and absence in CSF, and using white-blood-cell count in CSF. Patients received 1800 mg fexinidazole once per day on days 1–4 then 1200 mg fexinidazole on days 5–10. Patients were observed for approximately 19 months in total. Study participants were followed up on day 5 and day 8 during treatment, at end of treatment on day 11, at end of hospitalisation on days 11–18, at week 9 for a subset of patients, and after 6 months, 12 months, and 18 months. The primary endpoint was treatment success at 12 months. Safety was assessed through routine monitoring. Analyses were done in the intention-to-treat population. The acceptable success rate was defined as treatment efficacy in more than 80% of patients. This study is completed and registered with ClinicalTrials.gov (NCT02169557).

**Findings:**

Patients were enrolled between April 30, 2014, and April 25, 2017. 238 patients were recruited: 195 (82%) patients with stage 1 g-HAT and 43 (18%) with early stage 2 g-HAT. 189 (97%) of 195 patients with stage 1 g-HAT and 41 (95%) of 43 patients with early stage 2 g-HAT were finally included and completed the 10 day treatment period. Three patients with stage 1 g-HAT died after the 10 day treatment period and before the 12 month primary follow-up visit, considered as treatment failure and were withdrawn from the study. Treatment was effective at 12 months for 227 (99%) of 230 patients (95% CI 96·2–99·7): 186 (98%) of 189 patients (95·4–99·7) with stage 1 and 41 (100%) of 41 patients (91·4–100·0) with early stage 2, indicating that the primary study endpoint was met. No new safety issues were observed. The most frequent adverse events were headache and vomiting. In total, 214 (93%) of 230 patients had treatment-emergent adverse events, mainly common-terminology criteria for adverse events grades 1 to 3. None led to treatment discontinuation.

**Interpretation:**

Fexinidazole is a valuable first-line treatment option in the early stages of g-HAT.

**Funding:**

Through the Drugs for Neglected Diseases *initiative*: the Bill & Melinda Gates Foundation, the Republic and Canton of Geneva (Switzerland), the Dutch Ministry of Foreign Affairs (also known as DGIS; Netherlands), the Norwegian Agency for Development Cooperation (also known as Norad; Norway), the Federal Ministry of Education and Research (also known as BMBF) through KfW (Germany), the Brian Mercer Charitable Trust (UK), and other private foundations and individuals from the HAT campaign.

**Translation:**

For the French translation of the abstract see Supplementary Materials section.

## Introduction

Human African trypanosomiasis (HAT; sleeping sickness) caused by the parasite *Trypanosoma brucei gambiense* (g-HAT) is a potentially fatal neglected tropical disease.^1^ HAT has two clinical stages, the first (haemolymphatic stage 1) is associated with non-specific symptoms (intermittent fever, headache, pruritus, and lymphadenopathy).^1^ After 1 year or more, a second stage (meningoencephalitic stage 2) starts when trypanosomes cross the blood–brain barrier and invade the CNS resulting in disturbance of the sleep–wake cycle and neuropsychiatric signs and symptoms. Early stage 2 is characterised by a small increase in white blood cells (WBCs) in the cerebrospinal fluid (CSF; 6–20 WBCs/μL of CSF) and eventually evolves to late stage 2 (>20 WBCs/μL of CSF). Without appropriate diagnosis and treatment, the condition can cause permanent neurological damage and usually progresses to coma, organ failure, and death.^1^

Most reported g-HAT cases (70% in 2019) are diagnosed and treated in the Democratic Republic of the Congo,^2^ where this study was done. Although g-HAT remains endemic in sub-Saharan Africa, control programmes and chemotherapeutic treatment strategies have considerably lowered the prevalence of g-HAT cases worldwide.^3^, ^4^ The lowest number of new cases (n=864) since the start of systematic global data collection was reported in 2019.^2^

Stage 1 g-HAT has been treated with pentamidine since 1937, usually as a single daily intramuscular injection for 7 days. However, because pentamidine does not cross the blood–brain barrier efficiently enough to eliminate trypanosomes in the CNS, it is not used against stage 2 g-HAT.^2^ First-line therapy for stage 2 g-HAT is the highly effective nifurtimox–eflornithine combination therapy (NECT), but this treatment requires intravenous administration of eflornithine for 7 days, which is labour intensive and requires hospitalisation.

Treatment strategies have resulted in low g-HAT prevalence, but better tools that are safe, cheap, orally administered, and effective across all stages of g-HAT infection, without the need for CSF-based disease staging, would enable a simplified treatment that is easier to administer and follow. The development of fexinidazole, a new oral-only regimen, has filled an unmet need in g-HAT control.

The present study was done at sites where the pivotal trial was being done, facilitating comparison given that this meant both populations were similar. The pivotal trial was a phase 2 and 3 randomised, non-inferiority trial (NCT01685827) that showed that fexinidazole is effective and safe in patients with late stage 2 g-HAT.^5^ The present study aims to show that fexinidazole treatment also benefits patients with stage 1 or early stage 2 g-HAT.

**Research in context**

**Evidence before this study**

Human African trypanosomiasis (HAT) is a neglected tropical disease that can be fatal without appropriate diagnosis and treatment. The disease in humans is characterised by two clinical stages, depending on the localisation of infective *Trypanosoma* parasites, haemolymphatic stage 1 and meningoencephalitic stage 2, when trypanosomes cross the blood–brain barrier. The most common treatment previously available for stage 1 HAT caused by *Trypanosoma brucei gambiense* (g-HAT), which is the more prevalent form, was a 7 day course of intramuscular pentamidine, but this drug does not adequately cross the blood–brain barrier and is ineffective against stage 2 g-HAT. This disease stage has been treated with nifurtimox–eflornithine combination therapy (NECT), requiring intravenous administration of eflornithine two times per day for 7 days plus oral administration of nifurtimox three times per day for 10 days, which is a labour-intensive and inconvenient procedure in health structures of disease-endemic countries, such as the Democratic Republic of the Congo, where this study was done. Additionally, disease staging requires a lumbar puncture to assess cerebrospinal fluid (CSF) infection. A pivotal study established fexinidazole as the first oral monotherapy effective against stage 2 g-HAT. Its slightly lower success rate compared with NECT (91% *vs* 98% at 18 months) is potentially compensated for by an increased access to treatment.

**Added value of this study**

Our study is a plug-in to a pivotal trial that showed that fexinidazole is effective and safe compared with NECT in adult patients with late-stage g-HAT. With overall treatment success in 227 (99%) of 230 patients (95% CI 96·2–99·7) at 12 months, and 225 (98%) of 230 patients (95·0–99·3) at 18 months, the present study shows that fexinidazole also benefits adult and adolescent patients with stage 1 and early stage 2 g-HAT, and consolidates the role of oral fexinidazole as a valuable first-line treatment option in early g-HAT.

**Implications of all the available evidence**

Fexinidazole is a new g-HAT treatment option that is safe, easily administered, and effective across all stages of infection. This simplified oral regimen fills an unmet need in the control of HAT. With fexinidazole, treatment can be extended to most patients with g-HAT regardless of disease stage. Given that fexinidazole shows efficacy across disease stages, invasive lumbar punctures and CSF examinations might no longer be necessary for disease staging and treatment decisions in most patients. Oral administration and the fact that painful lumbar punctures would not be compulsory for disease staging mean that fewer staff and less equipment would be required. Treating patients with g-HAT with fexinidazole will be part of the efforts to achieve the WHO target of eliminating *T brucei gambiense* by 2030.

## Methods

### Study design and participants

For this prospective, multicentre, open-label, single-arm cohort study we recruited patients from eight treatment centres in the Democratic Republic of the Congo. The study included patients older than 15 years, who were able to ingest at least one complete meal per day (or at least one sachet of Plumpy'Nut (Nutriset, Malaunay, France), and who had a Karnofsky score higher than 50. Patients also had to have evidence of trypanosomes in their blood or lymph but no evidence of trypanosomes in their CSF. Patients were classified into stage 1 or early stage 2 g-HAT. Stage 1 g-HAT is defined by evidence of trypanosomes in the blood or lymph, no trypanosomes in the CSF, and 5 WBCs per μL CSF or less. The definition of early stage 2 g-HAT used in this study differs from that of stage 1 by the CSF WBC count only (6–20 WBCs/μL CSF). Patients with trypanosomes in the CSF were proposed for participation in the pivotal trial, irrespective of the number of CSF WBCs. Stage 1 or early stage 2 g-HAT with confirmed evidence of parasite in the blood or lymph, and absence of parasite in the CSF, was certified by reports from mobile teams (detailing examinations done and CSF WBC count) or done at the investigational site. Further inclusion criteria included willingness to be admitted to hospital to receive treatment, having a permanent address, and being able to comply with the follow-up visit schedule.

Exclusion criteria included severe malnutrition (body-mass index <16 kg/m^2^), inability to take medication orally, pregnant or breastfeeding women, any clinically important medical condition that could jeopardise patient safety or participation in the study, severely deteriorated general status, any contraindication to imidazole drugs, HAT treatment in the past 2 years, previous enrolment in the study or previous intake of fexinidazole, abnormalities on electrocardiogram (ECG) that did not return to normal in pretreatment repeated assessments or were considered clinically important, QT interval corrected using Fridericia's formula of at least 450 ms (on automatic reading on two successive ECGs in resting position, done 10–20 min apart), and patients not tested for malaria or not having received appropriate treatment for malaria or for soil-transmitted helminthiasis.

The study protocol was approved by independent ethics committees and regulatory authorities (Comité d'Ethique and Direction de la Pharmacie et des Medicaments, Ministry of Health of the Democratic Republic of the Congo, and the Comité de Protection des Personnes, Hôpital Necker, Paris, France). The study was designed and done in accordance with the Helsinki declaration and the International Council for Harmonisation E6 Good Clinical Practice Guidelines. All participants gave individual voluntary written informed consent. An independent data safety and monitoring board reviewed the study data regularly.

### Procedures

Over a 10 day treatment period, patients received 1800 mg (three 600 mg tablets) fexinidazole once per day for 4 days (loading phase, days 1–4), followed by 1200 mg (two 600 mg tablets) fexinidazole for 6 days (maintenance phase, days 5–10) within 30 min of the start of the main meal of the day, provided in the early morning.

Interruption of treatment was permitted for a maximum of 1 day with reintroduction at the investigator's discretion and an additional day of treatment added to make up for the missed dose. Treatment was to be discontinued in the following cases: severe skin reaction; isolated alanine aminotransferase (ALT) or aspartate aminotransferase (AST) higher than eight times the upper limits of normal (ULN); isolated ALT or AST higher than three times the ULN plus total bilirubin higher than two times the ULN; isolated ALT or AST higher than three times the ULN accompanied by fatigue, nausea, vomiting, right-upper-quadrant pain or tenderness, fever, rash with or without eosinophilia (>5% of all white blood cells in the blood); or any condition that, in the opinion of the investigator, required treatment discontinuation for medical reasons.

Patients were observed for approximately 19 months in total, from prescreening to an 18 month follow-up visit. Study participants were followed up on day 5 and day 8 during treatment, at end of treatment on day 11, at end of hospital treatment on days 11–18, at week 9 for a subset of patients (added as a safety precaution after a protocol amendment), and after 6 months, 12 months, and 18 months. The number of WBCs in the CSF was identified as a confounder in patients with stage 2 g-HAT, but not in patients with early-stage disease because the number of WBCs is capped by the inclusion criteria. The other known confounder was study centre. This source of heterogeneity was assessed through stratification, but in the absence of a comparator and knowledge of the real weight of each centre, there was no way to address the potential bias linked to the site effect, except giving the same weight to each centre in the estimation of success rate.

### Outcomes

In the pivotal study, fexinidazole was considered to be an acceptable treatment compared with NECT, with an acceptability margin of the CI of 13%, based on a survey of clinicians specialised in HAT; this acceptability margin is large but acceptable because the drug is more convenient to administer.^5^, ^6^ In the present study, an 80% success rate was considered to be unacceptable, calculated by subtracting the margin of 13% from the success rate expected with the reference treatments at 12 months (93%). The primary objective, as analysed by intention to treat (ITT), was therefore to show that the success rate of treatment with fexinidazole at 1 year follow-up in patients with stage 1 or early stage 2 g-HAT is greater than 80%. The primary efficacy endpoint was treatment outcome (effective or non-effective treatment), as assessed at the test-of-cure visit, 12 months after end of treatment. The outcome at 12 months was more conservative than the efficacy criteria proposed by WHO (appendix 2 p 1).^7^

Secondary objectives included the following: verifying whether the success rate of fexinidazole treatment varied depending on disease stage and, if the difference between stages was significant, verifying that the success rate for each stage was greater than 80% and compatible with the historical success rate reported with NECT (patients with stage 2 g-HAT) or pentamidine (patients with stage 1 g-HAT); verifying whether the success rate of fexinidazole treatment depends on WBC count in CSF before treatment initiation; studying the changes in success rate over time; and assessing the safety profile of fexinidazole and verifying whether this profile was similar to historical safety data for pentamidine.^8^

The secondary efficacy endpoint investigated treatment outcome at 24 h, 6 months, and 18 months after end of treatment, and descriptive safety analyses were done in the ITT population (in this study the ITT population is the same as patients who completed treatment). Adverse events were graded using NCI CTCAE, version 4.03.^9^ Adverse events reported by the patient or noted by the investigator, HAT signs and symptoms, ECGs, physical and neuropsychiatric examination, vital signs, standard haematology, and blood chemistry were also recorded.

A specific analysis was done on five adverse events of interest, including vomiting, neutropenia, anxiety, depression, and headache. These adverse events were selected a posteriori, for the following reasons: to check whether vomiting after treatment administration had any effect on compliance and to determine its timing; to analyse neutropenia, anxiety, and depression, which have been seen in patients with Chagas disease;^10^ and to analyse the incidence of headache, which has been seen in healthy volunteers.^11^

### Statistical analysis

On the basis of an expected success rate of 91%, we initially set the sample size to a minimum of 101 patients per stratum (stage 1 and early stage 2 g-HAT), giving at least 202 patients and a maximum of 300 patients overall. However, due to the small proportion of patients with early stage 2 g-HAT, the objective was only reached for patients with stage 1 g-HAT. A minimum sample size of 113 patients overall would have had a statistical power of at least 85%.

In the primary efficacy analysis, the success rate at 12 months was computed with its two-sided 95% CI using the Clopper-Pearson method. The lower limit of the CI was compared with the unacceptable rate of 80%. The primary analysis was not stratified by site, but any variation between sites was tested with an exact test in the ITT population.

The time course of the treatment success rate was plotted as a Kaplan-Meier curve. Success rate at each timepoint was compared by Cochran Q test. A logistical model for repeated measures based on the GLIMMIX SAS procedure was used to assess the effect of time on success rate, adjusting for the effect of study site. The relationship between the success rate at 12 months and WBC count in CSF at screening was estimated through logistic regression, with WBC count set as a quantitative covariate, and study site as a qualitative covariate. Missing values were not replaced except for the primary efficacy variable; we described the method of imputation used in the absence of the 12 month assessment visit (appendix 2 p 2). All summaries and statistical analyses were generated using the SAS software, version 9.2 or higher.

Methods of analysis of historical success rate reported for pentamidine in patients with stage 1 g-HAT, and the historical safety data for pentamidine are described in the appendix 2 (p 3).

This study is registered with ClinicalTrials.gov (number NCT02169557). The protocol is available online.^12^

### Role of the funding source

The funder of the study had no role in the study design, data collection, data interpretation, or writing of this report. The corresponding author had full access to all the data in the study and had final responsibility for the decision to submit for publication.

## Results

Patients were enrolled between April 30, 2014, and April 25, 2017. 238 patients were recruited, 195 (82%) patients with stage 1 g-HAT and 43 (18%) with early stage 2 g-HAT. Six patients with stage 1 g-HAT and two with early stage 2 group were subsequently excluded. 189 (97%) of 195 patients with stage 1 g-HAT and 41 (95%) of 43 patients with early stage 2 g-HAT completed the 10 day treatment period (figure).

**Figure:**
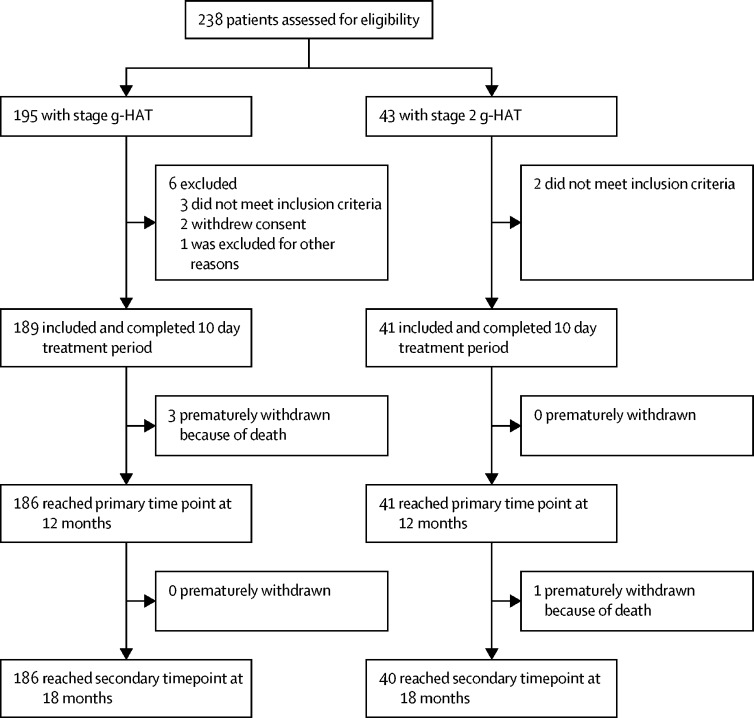
Study flowchart g-HAT=African trypanosomiasis caused by *Trypanosoma brucei gambiense*.

Baseline demographic characteristics were similar for patients with stage 1 or early stage 2 g-HAT (table 1). Medical history and clinical presentation at inclusion were consistent with disease stage. Mean WBC count was 3·97 cells per μL CSF (SD 3·41) overall (2·68 cells/μL CSF, SD 3·41, in patients with stage 1 and 9·88 cells/μL CSF, SD 4·00, in those with early stage 2).

**Table 1:** Baseline characteristics (ITT population)

		**Stage 1 (n=189)**	**Early stage 2 (n=41)**	**Total (n=230)**
Demographics				
	Men	93 (49%)	22 (54%)	115 (50%)
	Women	96 (51%)	19 (46%)	115 (50%)
	Age, years	34·16 (15·72)	35·15 (14·37)	34·33 (15·46)
	Weight, kg	49·59 (8·39)	53·26 (8·27)	50·24 (8·47)
	BMI, kg/m^2^	19·09 (2·16)	19·98 (2·43)	19·25 (2·23)
Parasitological findings*				
	Lymph examination	54 (29%)	15 (37%)	69 (30%)
	Positive lymph node	42 (22%)	12 (29%)	54 (23%)
	Blood examination	189 (100%)	41 (100%)	230 (100%)
	Positive CATT	178 (94%)	40 (98%)	218 (95%)
	Positive HAT RDT	11 (6%)	2 (5%)	13 (6%)
	Positive thick-blood smear	0	1 (2%)	1 (<1%)
	Positive CTC (Woo)	78 (41%)	10 (24%)	88 (38%)
	Positive mAECT	50 (26%)	15 (37%)	65 (28%)
	Positive mAECT-BC	25 (13%)	5 (12%)	30 (14%)
	CSF examination	189 (100%)	41 (100%)	230 (100%)
Method used to test for trypanosomes in the CSF				
	Direct	58 (31%)	13 (32%)	71 (31%)
	Single centrifugation	127 (67%)	27 (66%)	154 (67%)
	Unknown	4 (2%)	1 (2%)	5 (2%)
CSF WBC count, cells/μL		2·68 (1·23)	9·88 (4·00)	3·97 (3·41)
Vital signs and general health				
	SBP, mm Hg	108·46 (13·62)	107·93 (10·90)	108·36 (13·16)
	DBP, mm Hg	68·94 (9·04)	68·41 (7·28)	68·85 (8·74)
	Heart rate, bpm	77·27 (10·78)	75·20 (7·74)	76·90 (10·32)
	Respiratory rate per minute	19·80 (2·32)	19·49 (1·49)	19·75 (2·19)
	Temperature, °C	36·54 (0·43)	36·31 (0·44)	36·50 (0·44)
	General health-altered	5 (3%)	2 (5%)	7 (3%)

Data are presented as n (%) or mean (SD). BMI=body-mass index. bpm=beats per minute. CATT=card agglutination test for trypanosomiasis. CSF=cerebrospinal fluid. CTC (Woo)=capillary tube centrifugation by Woo test. DBP=diastolic blood pressure. HAT=human African trypanosomiasis. ITT=intention to treat. mAECT=mini-anion exchange centrifugation technique. mAECT-BC=mini-anion exchange centrifugation technique buffy coat. SBP=systolic blood pressure. RDT=rapid diagnostic test. WBC=white blood cell.

* Patients had CATT or RDT tests or had both tests. Patients not diagnosed by lymph-node aspiration, blood smear, or CTC had either mAECT-BC or mAECT tests.

In the ITT population, four patients died during follow-up and were prematurely withdrawn from the study. Of these, three patients with stage 1 g-HAT died after the 10 day treatment period and before the 12 month primary follow-up visit; therefore, in this cohort 186 (98%) of the 189 patients included in the study reached the primary timepoint at 12 months. One patient with early stage 2 g-HAT died between the 12 month and 18 month follow-up visits; therefore, in this cohort 40 (98%) of 41 patients reached the secondary timepoint at 18 months (figure).

Treatment was effective at 12 months for 227 (99%) of 230 patients (95% CI 96·2–99·7): 186 (98%) of 189 patients (95·4–99·7) with stage 1 and 41 (100%) of 41 patients (91·4–100·0) with early stage 2, indicating that the primary study endpoint was met (table 2). Not only was the lower limit of the 95% CI for treatment efficacy greater than 80%, it was also greater than the expected efficacy rate of 91% based on the results of the pivotal study,^5^ which accords with the patients having less severe forms of g-HAT in this study. We did not find any statistically significant variation in the overall success rate at 12 months between investigational sites.

**Table 2:** Success rate at 12 months and 18 months (ITT population)

	**Stage 1 (n=189)**		**Early stage 2 (n=41)**		**Total (n=230)**	
	N (%)	95% CI	N (%)	95% CI	N (%)	95% CI
Response to treatment at 12 months	186 (98%)	95·4–99·7	41 (100%)	91·4–100·0	227 (99%)	96·2–99·7
Did not respond to treatment at 12 months	3 (2%)	0·3–4·6	0	0–8·6	3 (1%)	0·3–3·8
Response to treatment at 18 months	185 (98%)	94·7–99·4	40 (98%)	87·1–99·9	225 (98%)	95·0–99·3
Did not respond to treatment at 18 months	4 (2%)	0·6–5·3	1 (2%)	0·1–12·9	5 (2%)	0·7–5·0

ITT=intention to treat.

At 12 months, three patients did not respond to treatment in the ITT population (all three with stage 1 g-HAT; appendix 2 p 4). These patients died from causes unrelated to treatment or disease (ie, meningeal syndrome and encephalitis, reported 40 days after end of treatment with death 1 week after the start of symptoms; shock, reported 191 days after end of treatment, with death 1 day thereafter; and peritonitis in a patient with a background of Crohn's disease, which was diagnosed by biopsy and reported after 143 days and death 335 days after end of treatment). No statistically significant relationship was found between the 12 month success rate and the WBC count in CSF at screening, and due to the low non-response rates, no significant difference in treatment efficacy over time was detected.

At 18 months, treatment was effective in 225 (98%) of 230 patients (95% CI 95·0–99·3): 185 (98%) of 189 patients (94·7–99·4) with stage 1 g-HAT and 40 (98%) of 41 patients (87·1–99·9) with early stage 2 g-HAT. A fourth death occurred at home in a patient with early stage 2 g-HAT with decompensated anaemia, sepsis of pulmonary origin, and probable nephropathy (422 days after the end of treatment). Another patient with stage 1 g-HAT, in whom treatment was effective at 12 months, was reclassified as not responding to treatment, because their WBCs were higher than 20 cells per μL CSF at 18 months, although no rescue treatment was administered because meningitis was diagnosed and treated with antibiotics (a 22 month supplementary visit confirmed full recovery). Another patient with early stage 2 g-HAT died after the 18 month visit due to cardiogenic shock and hypovolaemic shock (551 days after end of treatment) and was, therefore, not included in the analysis.

Historical non-response to pentamidine treatment (4·6–9·2%)^13^, ^14^ is higher than that obtained in the present study, in which three (2%) of 189 patients with stage 1 g-HAT did not respond to treatment within 12 months. Historical non-response to NECT is not evaluable because the populations treated with NECT were different; however, 41 patients with early stage 2 HAT did not respond to fexinidazole treatment.

We assessed signs and symptoms of HAT as an evaluation of treatment efficacy (appendix 2 p 5) and we noted an overall improvement at end of hospital treatment and 18 months. The overall incidence of clinical signs and symptoms of HAT decreased during the study, and at end of hospital treatment we observed a substantial decrease in the symptoms most frequently reported at inclusion, particularly headache (from 154 [67%] of 230 patients to 13 [6%] of 229), weight loss (from 83 [36%] of 229 patients to 31 [13%] of 229), fever (from 58 [25%] of 230 patients to one [<1%] of 229), asthenia (from 53 [23%] of 229 patients to nine [4%] of 229), and pruritus (from 51 [22%] of 230 patients to six [3%] of 229). Despite some fluctuations after end of hospital treatment, the reduction in incidence of clinical signs and symptoms of HAT was maintained during the 18 month follow-up: headache was still present in 16 (7%) of 226 patients, weight loss in ten (4%) of 226, fever in nine (4%) of 226, asthenia in six (3%) of 226, and pruritus in six (3%) of 226.

In total, 214 (93%) 230 patients had 1261 treatment-emergent adverse events (TEAEs) during the study: 176 [93%] of 189 patients with stage 1 g-HAT had 1002 TEAEs and 38 [93%] of 41 patients with early stage 2 g-HAT had 259 TEAEs (table 3; appendix 2 p 6). Most were mild or moderate (in total 1219 [97%] of 1261 TEAEs; 970 [97%] of 1002 TEAEs in patients with stage 1 g-HAT and 249 [96%] of 259 TEAEs in those with early stage 2 g-HAT) and considered possibly related to treatment (in total 860 [68%] of 1261 TEAEs; 683 [68%] of 1002 TEAEs in patients with stage 1 g-HAT and 177 [68%] of 259 TEAEs in those with early stage 2 g-HAT). Treatment-emergent serious adverse events were reported in 22 patients. Only one serious adverse event (SAE; psychotic disorder) started during the 10 day treatment period, and only two SAEs in 230 patients were considered to be possibly treatment related (blood sodium decrease and psychotic disorder). No TEAEs led to temporary or permanent study-drug discontinuation. The most frequently reported TEAEs (in >5% of patients) are presented in table 4. Gastrointestinal adverse events were the most frequently reported events overall. Despite a reasonably high rate of vomiting, treatment was only readministered in 15 (7%) of 230 patients, because most patients who vomited did so more than 60 min after treatment administration. Concomitantly, 28 (12%) of 230 patients received anti-emetic drugs. No severe cases were reported. No adverse events led to treatment discontinuation. None of the events leading to the four deaths were established to be related to treatment, and all were considered unexpected for fexinidazole. Psychiatric and neurological examination did not reveal any new safety signals in relation to the pivotal trial. Insomnia and tremor occurred in approximately a quarter of patients. One severe and serious event of psychotic disorder occurred during treatment and was considered to be possibly related to fexinidazole.

**Table 3:** Incidence of TEAE and SAEs at 18 months in the ITT population

	**Number of patients (N=230)**	**Number of events**
At least one adverse event	214 (93%)	1280
At least one TEAE	214 (93%)	1261
At least one TEAE during the treatment period	212 (92%)	1166
At least one TEAE after the treatment period	63 (27%)	95
At least one TEAE leading to treatment discontinuation (temporary or permanent)	0	..
At least one TEAE leading to permanent treatment discontinuation	0	..
At least one mild or moderate TEAE	214 (93%)	1219
At least one severe TEAE	28 (12%)	41
At least one TEAE possibly related to treatment	195 (85%)	860
At least one SAE	23 (10%)	38
At least one TESAE	22 (10%)	34
At least one TESAE during the treatment period	1 (<1%)	1
At least one TESAE after treatment period	21 (9%)	33
At least one TESAE leading to treatment discontinuation (temporary or permanent)	0	..
At least one TESAE leading to permanent treatment discontinuation	0	..
At least one TESAE possibly related to treatment	2 (1%)	2
At least one TESAE leading to death	4 (2%)	4

ITT=intention to treat. SAE=serious adverse event. TEAE=treatment-emergent adverse event. TESAE=treatment-emergent serious adverse event.

**Table 4:** Summary of treatment-emergent adverse events reported in at least 5% of patients compared with the pivotal study at 18 months in the intention-to-treat population^6^

		**Current study**		**Pivotal study**	
		n (%) of patients (N=230)	n (%) of adverse events	Number of patients (N=264)	Number of adverse events
Any treatment-emergent adverse events		214 (93%)	1261	247 (94%)	1525
Gastrointestinal disorders		179 (78%)	427	157 (59%)	353
	Nausea	99 (43%)	125	75 (28%)	101
	Vomiting	97 (42%)	114	68 (26%)	73
	Dyspepsia	46 (20%)	52	34 (13%)	43
	Upper abdominal pain	22 (10%)	27	27 (10%)	32
	Gastritis	17 (7%)	24	8 (3%)	13
	Salivary hypersecretion	16 (7%)	20	16 (6%)	17
	Abdominal pain	17 (7%)	19	25 (9%)	29
Nervous-system disorders		142 (62%)	283	158 (60%)	308
	Headache	93 (40%)	134	92 (35%)	134
	Dizziness	58 (25%)	74	50 (19%)	56
	Tremor	52 (23%)	61	58 (22%)	68
General disorders and administration-site conditions		94 (41%)	151	122 (46%)	184
	Asthenia	70 (30%)	86	60 (23%)	73
	Feeling hot	38 (17%)	48	25 (9%)	29
	Pyrexia	3 (1%)	4	23 (9%)	27
	Chest pain	4 (2%)	4	23 (9%)	25
Psychiatric disorders		73 (32%)	110	10 (4%)	159
	Insomnia	57 (25%)	69	74 (28%)	83
	Hallucinations	12 (5%)	14	3 (1%)	3
Metabolism and nutrition disorders		48 (21%)	58	131 (50%)	190
	Decreased appetite	44 (19%)	52	56 (21%)	58
	Hypocalcaemia	1 (<1%)	1	36 (14%)	37
Musculoskeletal and connective tissue disorders		38 (17%)	53	58 (22%)	92
	Back pain	17 (7%)	21	30 (11%)	36
	Neck pain	15 (7%)	18	23 (9%)	27
Investigations		41 (18%)	49	7 (3%)	8
	Blood sodium decreased	16 (7%)	17	20 (8%)	21
	Blood potassium increased	11 (5%)	11	27 (10%)	29
	Blood albumin decreased	7 (3%)	7	23 (9%)	24
Vascular disorders		18 (8%)	20	24 (9%)	26
	Hot flush	12 (5%)	13	13 (5%)	13
Cardiac disorders		17 (7%)	18	18 (7%)	21
	Palpitations	14 (6%)	15	13 (5%)	16
Eye disorders		16 (7%)	17	15 (6%)	18
Blood and lymphatic-system disorders		14 (6%)	15	29 (11%)	33
	Anaemia	7 (3%)	7	24 (9%)	25
Infections and infestations		14 (6%)	16	22 (8%)	33
Skin and subcutaneous tissue disorders		13 (6%)	15	22 (8%)	23
Respiratory, thoracic, and mediastinal disorders		9 (4%)	12	32 (12%)	34
	Cough	4 (2%)	4	16 (6%)	16
Injury, poisoning, and procedural complications		15 (6%)	18	14 (15%)	20
	Wound	1 (<1%)	1	9 (3%)	9

Of the five adverse events of interest that were analysed separately, the most frequently observed during hospital admission were vomiting and headache. Neutropenia was reported in four patients during hospital treatment, which was severe in one patient. Other adverse events of interest were rare (such as anxiety, which occurred in one patient), or did not occur, such as depression. We did not record any SAEs related to neutropenia or either acute or delayed liver abnormalities during the study. The risks of delayed neutropenia and delayed-onset increase in liver aminotransferases, observed at higher total fexinidazole doses in patients with Chagas disease (minimum 27 g fexinidazole),^8^ were not confirmed at the total dose administered in the present study (14·4 g fexinidazole). The safety profile of fexinidazole in terms of percentage of patients reported with TEAEs, related TEAEs, and SAEs can be considered similar to that of pentamidine.

## Discussion

This prospective, multicentre, open-label, single-arm cohort study was a plug-in to the pivotal study,^5^ as aforementioned, and was designed to assess the efficacy and safety of fexinidazole in stage 1 and early stage 2 g-HAT. The primary efficacy endpoint of successful treatment outcome 12 months after end of treatment was met, and results exceeded expectations. The efficacy of fexinidazole was high at both 12 month and 18 month visits.^5^ Our study consolidates the role of oral fexinidazole in g-HAT, showing that this drug is also a valuable first-line treatment option in early stages of disease. In this study, the limit of unacceptable success rate for fexinidazole treatment at 12 months was set to 80%. The efficacy results were non-inferior to the historical results of pentamidine among patients with stage 1 g-HAT (95·4–91·8%).^13^, ^14^

Comparing the threshold WBC count in CSF between stage 1 and stage 2 HAT is challenging. In most national programmes and in the present study, a threshold of 5 WBCs per μL of CSF was set to distinguish stage 1 and stage 2 g-HAT (in the absence of trypanosomes in the CSF), whereas a threshold of 20 WBCs per μL was used in the pivotal study, and 10 WBCs per μL was used in another study.^8^ Moreover, the age group (adults only *vs* adults and children) and the duration of follow-up (6 months, 12 months, and up to 24 months)^15^ are relatively heterogeneous between studies.

Assessment of treatment success was highly conservative. Death, regardless of the cause, and possible loss to follow-up were considered to indicate that the treatment was not effective. The risk of observing false successes was decreased by the multiple approaches we used to assess post-treatment outcome (clinical, parasitological, and biological), and by technical supervision.

Had we applied WHO success criteria,^5^ we would not have detected any non-response to treatment and efficacy would have been 100%. The four reported deaths happened after 30 days, and the raised number of WBCs in the CSF (110 WBCs/μL CSF) at 18 months in one patient could be attributed to acute meningitis, which was treated with antibiotics and declared cured in an additional visit at 22 months.

As in the pivotal study, the present study was open label. There were no direct comparators; instead we used historical results for NECT and pentamidine, and there were no control groups, given that the underlying goal was to consolidate the results of the pivotal study and extend treatment with fexinidazole to all adult patients with g-HAT, regardless of disease stage. After consultation with the European Medicines Agency, the duration of follow-up for the primary-endpoint assessment was set at 12 months (6 months shorter than in the pivotal study) to expedite the granting of a scientific opinion, although this follow-up period reduces comparability with other studies that precisely follow the WHO methodological framework for HAT clinical trials. However, late treatment non-response between 12 months and 18 months was expected to be low (~1·2% based on historical data with melarsoprol, eflornithine,^16^ and NECT),^15^, ^17^ and an additional follow-up was done at 18 months after end of treatment (the actual treatment non-response rate between 12 months and 18 months was 0·9%).

Most of the reported adverse events were similar to the HAT signs and symptoms investigated at the point of patient inclusion. Distinguishing between disease-related symptoms, comorbidities, or study drugs is often challenging.^18^, ^19^ However, the appearance of HAT symptoms that were not present at baseline was rare, indicating that the signs and symptoms reported during the study were more likely to be a consequence of the disease than the result of an adverse drug reaction. The safety data collected during this study did not raise any new safety issues besides those already known, such as gastrointestinal (nausea and vomiting) and CNS-related adverse events, mainly headache.^5^ A larger number of TEAEs were detected in this study compared with the pivotal trial. In HAT trials, differentiating between TEAEs and signs and symptoms of the disease is difficult.^18^, ^19^ This trial enrolled patients with clinically less severe disease with less serious symptoms at baseline, which could have led to more reporting of TEAEs than in the pivotal trial, given that patients in the pivotal trial had pre-existing symptoms. The present study should, therefore, give better information about the true safety profile of fexinidazole, with the most frequent symptoms being nausea, vomiting, headache, asthenia, dizziness, insomnia, and tremor reported in more than one in five patients. The TEAE more frequently reported among patients who were more severely ill in the pivotal trial might be attributed to the disease itself and include back pain, increase in blood potassium, anaemia, decrease in blood albumin, chest pain, cough, pyrexia, or hypocalcaemia. The majority of adverse events were mild or moderate and none led to treatment discontinuation. Thus, fexinidazole's benefit–risk balance for treating adults with stage 1 or early stage 2 g-HAT is clearly positive.

Our study is limited by its single treatment group, and hence its open-label study design. Patients with this disease are rare and the number of patients available to take part in the trial would not have allowed for a sufficiently powered comparison with pentamidine for stage 1 g-HAT and NECT for stage 2 g-HAT. In addition, the differing modes of treatment administration would have made a double-blind comparative study impossible. However, bias towards a higher success rate was limited by applying stricter criteria than those proposed by WHO, given that any death and possible loss to follow-up are considered to indicate treatment inefficacy. To mitigate the risk of the overestimation of success, other criteria were also considered to indicate the treatment not being effective, and include: receiving rescue medication; trypanosomes in any body fluid; or raised numbers of WBCs in the CSF, independently of the cause, which are easy to measure objectively. Selection bias caused by the study target population that included patients with mild g-HAT, and the low numbers of patients with early stage 2, makes comparison with NECT unfeasible.

Potential concerns with respect to adherence are present, given that fexinidazole must be administered during or after a main meal to achieve effective concentrations, that the dosing schedule of 10 days is relatively long for an oral treatment, that the number of tablets changes midway through treatment, and that nausea and vomiting are frequent side-effects. A study including a subcohort on home-based treatment has recently been completed at the time of writing to help assess these difficulties (NCT03025789).

A simplified oral regimen affecting both disease stages rather than intravenous treatment overcomes obstacles to the integration of g-HAT care in peripheral health structures, particularly in areas with scarce health-care resources. The pivotal fexinidazole study established this drug to be the first oral monotherapy effective against stage 2 g-HAT. The slightly lower success rate of fexinidazole compared with NECT is compensated for by its ease of use and simplified access to treatment for patients and health-care systems, including supply logistics. Given that fexinidazole shows efficacy across disease stages, invasive lumbar punctures and CSF examinations are no longer necessary for all patients. The most recent WHO treatment guidelines state that a lumbar puncture is only required to confirm severe stage 2 HAT.^20^ Fexinidazole might contribute to achieving the WHO target of eliminating *T brucei gambiense* by 2030.^21^

## Data sharing

The data underlying the results of this study are available upon request because they contain potentially sensitive information. Interested researchers can contact the DND*i*, the commissioner of this study, for data access requests via email at CTdata@dndi.org. Researchers can also request data by completing the form available online. In this form, they confirm that they will share data and results with DND*i* and will publish any results in an open-access format.

## Declaration of interests

BS reports personal fees from the Drugs for Neglected Diseases *initiative* (DND*i*) during the conduct of the study and outside the submitted work. CB, OVM, DNT, SB, FS, SD, NS-W, and AT report employment at DND*i*. All other authors declare no competing interests.

## Supplementary Materials

French translation of the abstract
