# Supplementary material for: Oral fexinidazole for stage 1 or early stage 2 African Trypanosoma brucei gambiense trypanosomiasis: a prospective, multicentre, open-label, cohort study
Source: Lancet Glob Health. 2021 Jun 15;9(7):e999–e1008. doi: 10.1016/S2214-109X(21)00208-4 (PMC8220131; doi:10.1016/S2214-109X(21)00208-4)
Supplement: French translation of the abstract [file mmc1.pdf]

THE LANCET
Global Health

Supplementary appendix 1

This translation in French was submitted by the authors and we reproduce it as supplied. It has not been peer reviewed. *The Lancet*'s editorial processes have only been applied to the original in English, which should serve as reference for this manuscript.

Cette traduction en français a été proposée par les auteurs et nous l'avons reproduite telle quelle. Elle n'a pas été examinée par des pairs. Les processus éditoriaux du *Lancet* n'ont été appliqués qu'à l'original en anglais et c'est cette version qui doit servir de référence pour ce manuscrit.

Supplement to: Kande Betu Ku Mesu V, Mutombo Kalonji W, Bardonneau C, et al. Oral fexinidazole for stage 1 or early stage 2 African *Trypanosoma brucei gambiense* trypanosomiasis: a prospective, multicentre, open-label, cohort study. *Lancet Glob Health* 2021; **9:** e999–1007.

Fexinidazole orale pour la trypanosomiase africaine à *Trypanosoma brucei gambiense* au stade 1 ou au stade 2 précoce : étude de cohorte prospective, multicentrique et ouverte

Généralités

La détermination du stade et le traitement de la trypanosomiase humaine africaine due à *Trypanosoma brucei gambiense* (THA-g) nécessitaient une ponction lombaire pour analyser le liquide céphalo-rachidien (LCR), et l'administration par voie intraveineuse de médicaments qui traversent la barrière hémato-encéphalique pour traiter les infections à un stade avancé. Ces procédures sont peu pratiques pour les systèmes de santé en milieu rural dans les pays où la THA est endémique. Une étude pivot a établi que le fexinidazole est la première monothérapie orale efficace contre la THA-g au stade 2 non grave. Nous avons cherché à évaluer la sécurité et l'efficacité du fexinidazole pour la THA-g aux stades précoces.

Méthodes

Dans cette étude de cohorte prospective, multicentrique, ouverte et à un seul bras, des patients atteints de THA-g au stade 1 ou au stade 2 précoce ont été recrutés dans huit centres de traitement en République démocratique du Congo. Les principaux critères d'inclusion étaient les suivants : être âgé de plus de 15 ans, être capable d'ingérer au moins un repas complet par jour (ou au moins un sachet de Plumpy'Nut®), avoir un score de Karnofsky supérieur à 50, mise en évidence de trypanosomes dans le sang ou la lymphe mais pas dans le LCR, accepter d'être hospitalisé pour recevoir le traitement, avoir une adresse permanente, et être capable de respecter le calendrier des visites de suivi. Les critères d'exclusion comprenaient la malnutrition sévère, l'incapacité à prendre des médicaments par voie orale, grossesse ou allaitement pour les femmes, toute pathologie cliniquement importante pouvant compromettre la sécurité du patient ou sa participation à l'étude, un état général très détérioré, toute contre-indication aux imidazolés, des antécédents de traitement pour la THA au cours des 2 dernières années, une participation antérieure à l'étude ou une prise antérieure de fexinidazole, des anomalies électrocardiographiques non normalisées lors des évaluations répétées avant le traitement ou considérées comme cliniquement importantes, un intervalle QT corrigé selon la formule de Fridericia d'au moins 450 ms, et l'absence de test diagnostic du paludisme ou l'absence de traitement approprié pour le paludisme ou les helminthiases transmises par le sol. Les patients ont été classés en deux groupes, THA-g au stade 1 ou THA-g stade 2 précoce, en fonction de la présence de trypanosomes dans le sang, la lymphe et l'absence dans le LCR, et du taux de globules blancs dans le LCR. Les patients ont reçu 1800 mg de fexinidazole une fois par jour aux jours 1 à 4, puis 1200 mg de fexinidazole aux jours 5 à 10. Les patients ont été suivis pendant

environ 19 mois au total, avec une visite de suivi aux jours 5 et 8 pendant le traitement, à la fin du traitement au jour 11, à la fin de l'hospitalisation aux jours 11-18, à la semaine 9 pour un sous-groupe de patients, ainsi qu'aux mois 6, 12 et 18. Le critère d'évaluation principal était le succès du traitement à 12 mois. L'évaluation de la sécurité était basée sur la surveillance de routine. Les analyses ont été effectuées dans la population en intention de traiter. Le taux de réussite acceptable a été défini par l'efficacité du traitement chez plus de 80% des patients. Cette étude est terminée et enregistrée auprès de ClinicalTrials.gov (NCT02169557).

Conclusions

Les patients ont été recrutés entre le 30 avril 2014 et le 25 avril 2017. Au total 238 patients atteints de THA-g ont été recrutés, dont 195 (82%) au stade 1 et 43 (18%) au stade 2 précoce. Sur les 195 patients atteints de THA-g au stade 1, 189 (97%) ont été finalement inclus et terminé le traitement de 10 jours, et sur les 43 atteints de THA-g au stade 2 précoce, 41 (95%) ont terminé le traitement de 10 jours. Trois patients atteints de THA-g au stade 1 sont décédés après le traitement de 10 jours mais avant la visite de suivi à 12 mois, considérées comme échecs dans l'analyse, n'ayant pas complété l'étude. Le traitement était efficace à 12 mois chez 227 patients (99%) sur 230 (IC à 95% : 96·2–99·7), soit chez 186 patients atteints de THA-g au stade 1 (98%) sur 189 (IC à 95%: 95·4–99·7), et chez 41 patients atteints de THA-g au stade 2 précoce (100%) sur 41 (IC à 95% : 91·4–100·0), indiquant que le critère d'évaluation principal a été atteint. Aucun nouveau problème de sécurité n'a été mis en évidence. Les événements indésirables les plus fréquents étaient des céphalées et des vomissements. Des événements indésirables survenus pendant le traitement ont été observés chez 214 patients (93%) sur 230, ces événements correspondant principalement aux critères communs de terminologie pour les événements indésirables (CTCAE) de grade 1 à 3 Aucun événement n'a entraîné l'arrêt du traitement.

Interprétation

Le fexinidazole est une option thérapeutique de première intention précieuse pour les stades précoces de la THA-g.

Financement

Par l'intermédiaire de DND*i* (Drugs for Neglected Diseases initiative) : la Fondation Bill & Melinda Gates (BMGF), la République et Canton de Genève (Suisse), le Ministère Néerlandais des Affaires

Étrangères (également connu sous le nom de DGIS ; Pays-Bas), l'Agence Norvégienne de Coopération au Développement (également connue sous le nom de Norad ; Norvège), le Ministère Fédéral de l'Éducation et de la Recherche (également connu sous le nom de BMBF) par l'intermédiaire de KfW (Allemagne), le Brian Mercer Charitable Trust (Royaume-Uni), ainsi que d'autres fondations privées et des particuliers participant à la campagne contre la THA.
