# Supplementary material for: Oral fexinidazole for stage 1 or early stage 2 African Trypanosoma brucei gambiense trypanosomiasis: a prospective, multicentre, open-label, cohort study
Source: Lancet Glob Health. 2021 Jun 15;9(7):e999–e1008. doi: 10.1016/S2214-109X(21)00208-4 (PMC8220131; doi:10.1016/S2214-109X(21)00208-4)
Supplement: Supplementary appendix 2 [file mmc2.pdf]

THE LANCET
Global Health

Supplementary appendix 2

This appendix formed part of the original submission and has been peer reviewed. We post it as supplied by the authors.

Supplement to: Kande Betu Ku Mesu V, Mutombo Kalonji W, Bardonneau C, et al. Oral fexinidazole for stage 1 or early stage 2 African *Trypanosoma brucei gambiense* trypanosomiasis: a prospective, multicentre, open-label, cohort study. *Lancet Glob Health* 2021; **9:** e999–1007.

Supplementary Materials

Figure S1. Algorithm of classification to categorise treatment success for the primary efficacy endpoint (12 months)

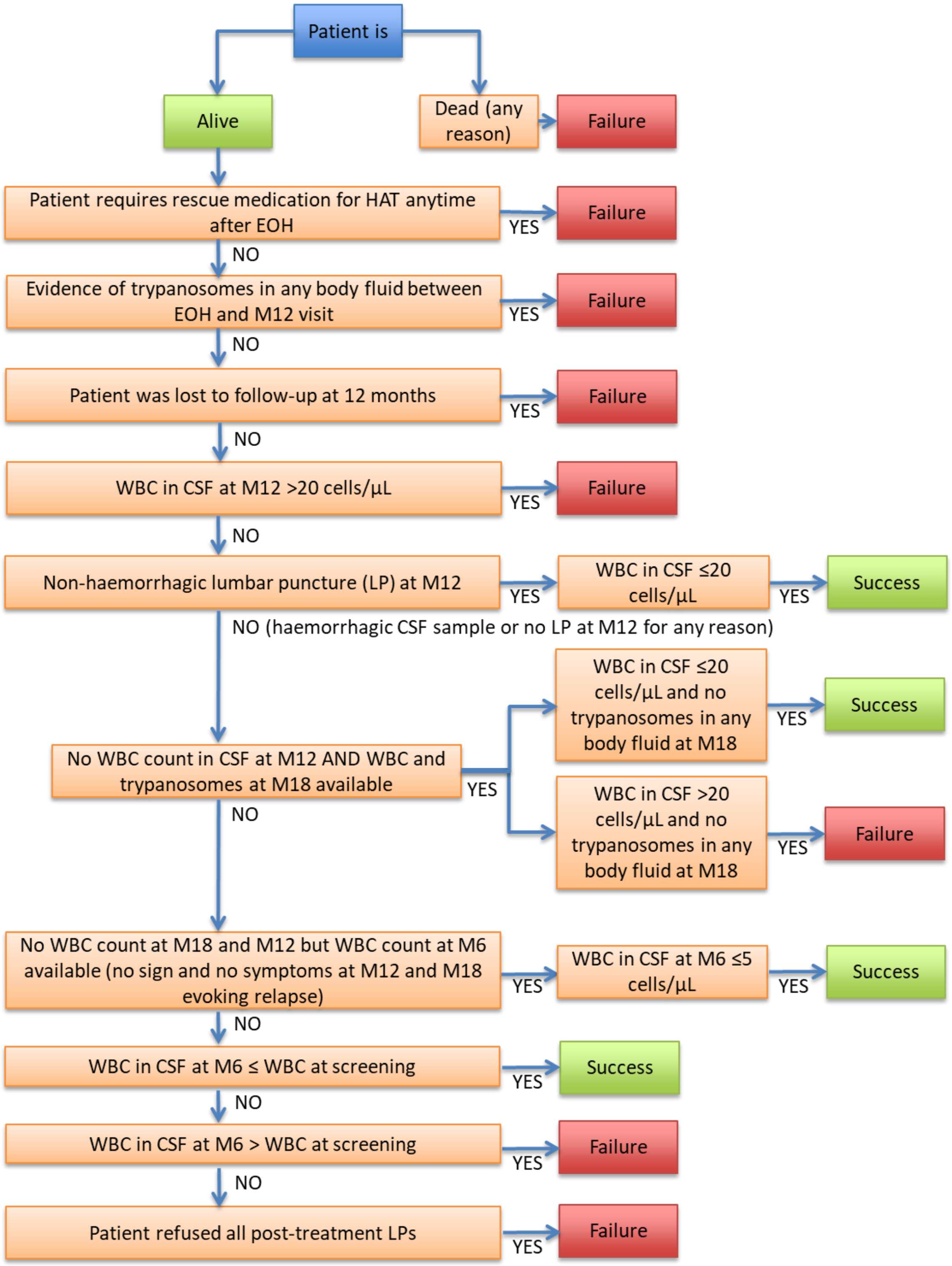

CSF, cerebrospinal fluid; EOH, end of hospitalisation; HAT, human African trypanosomiasis; LP, lumbar puncture; M, month; WBC, white blood cell count.

Table S2. Methods of imputation for missing data regarding the primary efficacy variable (outcome at 12 months)

Imputation method	Rules of imputation
Primary method	Probable success (considered as a success) if the patient attended the 12-month visit but refused the lumbar puncture at 12 months, showed no signs or symptoms of human African trypanosomiasis at 12 months, did not report any sign of relapse later, and had a favourable evolution at the last available assessment (18 months, or 6 months if the lumbar puncture was missing at 18 but not 6 months).<br>Otherwise, the outcome was considered as a failure.<br>The outcome for any patient lost to follow-up at 18 months or before was also imputed as a failure.<br>The algorithm of classification provided the status at 18 months for all patients.

Methods S3 Analysis of historical success rate reported for pentamidine in patients with stage 1 human African Trypanosomiasis, and the historical safety data for pentamidine

In a retrospective study of pentamidine in patients with stage 1 HAT, 23 failures (4.6%) were observed (N.B. only 371/652 patients were followed-up for 12 months).[1] In a large meta-analysis on 2,524 patients treated with pentamidine, relapse rates of 5.6%, 8.2%, and 9.2% were obtained in patients followed for 6, 12, or up to 18 months.[2] These failure rates are higher than that obtained in the present study (1.6%), with 3 failures observed within 12 months in the 189 patients who presented with stage 1 HAT. A Phase IIb study in 81 patients (41 treated with pentamidine) and a Phase III study in 273 patients (137 treated with pentamidine) with early stage HAT used pentamidine as a comparator treatment.[3,4] In the current study, TEAEs occurring during the treatment period with fexinidazole were reported in 92.2% of patients, compared to 93% of patients during treatment with pentamidine in the Phase IIb study and 98.5% of patients in the Phase III study. Adverse events considered at least possibly related to treatment were reported in 84.8% of patients in the current study (over the whole reporting period) compared with 92.7% of patients in the Phase III study (during the treatment period). In all 3 studies the majority of AEs were mild or moderate in severity. TESAEs were reported in 20/230 (8.7%) of patients in the ITT population in the current study, 24/137 patients (17.5%) in the Phase III study with pentamidine. None of the deaths in any study were considered related to treatment. The safety profile of fexinidazole in terms of percentage of patients reported with TEAEs, related TEAEs and SAEs can be considered comparable to that of pentamidine.

1. Balasegaram M, Harris, S, Checchi F, Hamel C, Karunakara U. Treatment outcomes and risk factors for relapse in patients with early-stage human African trypanosomiasis (HAT) in the Republic of the Congo. *Bull World Health Organ* 2006;**84**:777-82.
2. Hümbelin M. Definition of follow-up duration in clinical research of human African trypanosomiasis. Thesis in pharmaceutical sciences at the University of Basel, Switzerland. 2006
3. Burri C, Yeramian PD, Allen J et al. Efficacy, Safety, and Dose of Pafuramidine, a New Oral Drug for Treatment of First Stage Sleeping Sickness, in a Phase 2a Clinical Study and Phase 2b Randomized Clinical Studies. *PLoS Negl Trop Dis* 2016;**10**:e0004362.
4. Pohlig G, Bernhard SC, Blum J et al. Efficacy and Safety of Pafuramidine versus Pentamidine Maleate for Treatment of First Stage Sleeping Sickness in a Randomized, Comparator-Controlled, International Phase 3 Clinical Trial. *PLoS Negl Trop Dis* 2016;**10**:e0004363.

Table S4. Summary of grade 5 (death) treatment emergent adverse events (**N=230)**

**Sex**	**Age**	**Start date – end date (days)***	**Any TEAE of grade 5**
Male	15	40-44	Encephalitis + meningeal disorder
Male	35	143-335	Peritonitis
Male	69	191-192	Shock
Female	27	422-422	Anaemia pulmonary sepsis + nephropathy
Female	43	551	Cardiogenic shock + hypovolaemic shock

*Number of days after last dose of fexinidazole that the event occurred

Figure S5. HAT clinical signs and symptoms over time: radar chart at inclusion and EOH (ITT population)

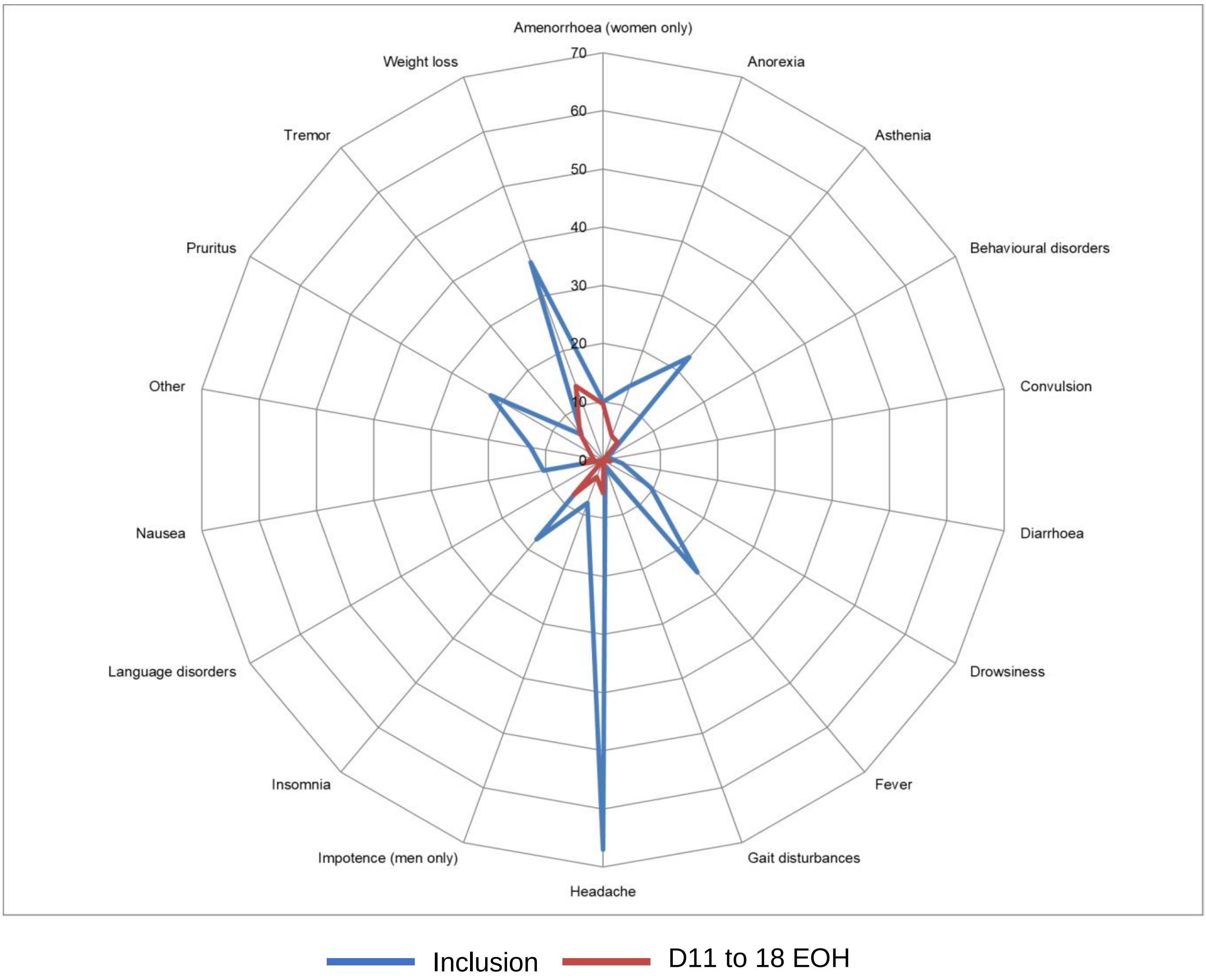

D, Day; EOH, end of hospitalisation; HAT, human African trypanosomiasis; ITT, intention-to-treat.

Table S6. Summary of any grade 3-4 treatment emergent adverse events (TEAEs) in the ITT population

	Grade 3<br>N=230	Grade 4<br>N=230	
**Any TEAE of grades 3-4**	**23 (10.0%) [31]**	**3 (1.3%) [3]**	**23 (11.3%) [34]**
Inguinal hernia	4 (1.7%) [7]	0 (0.0%) [0]	4 (1.7%) [7]
Blood potassium increased	4 (1.7%) [4]	0 (0.0%) [0]	4 (1.7%) [4]
Appendicitis	3 (1.3%) [3]	0 (0.0%) [0]	3 (1.3%) [3]
Blood sodium decreased	1 (0.4%) [1]	1 (0.4%) [1]	2 (0.9%) [2]
Headache	2 (0.9%) [2]	0 (0.0%) [0]	2 (0.9%) [2]
Ovarian cyst	2 (0.9%) [2]	0 (0.0%) [0]	2 (0.9%) [2]
Blood glucose increased	1 (0.4%) [1]	1 (0.4%) [1]	1 (0.4%) [2]
Anaemia	1 (0.4%) [1]	0 (0.0%) [0]	1 (0.9%) [1]
Crohn's disease	1 (0.4%) [1]	0 (0.0%) [0]	1 (0.4%) [1]
Inguinal hernia strangulated	1 (0.4%) [1]	0 (0.0%) [0]	1 (0.4%) [1]
Cerebral malaria	1 (0.4%) [1]	0 (0.0%) [0]	1 (0.4%) [1]
Pulmonary tuberculosis	1 (0.4%) [1]	0 (0.0%) [0]	1 (0.4%) [1]
Sciatica	1 (0.4%) [1]	0 (0.0%) [0]	1 (0.4%) [1]
Neutropenia	1 (0.4%) [1]	0 (0.0%) [0]	1 (0.4%) [1]
Dysuria	1 (0.4%) [1]	0 (0.0%) [0]	1 (0.4%) [1]
Malignant hypertension	0 (0.0%) [0]	1 (0.4%) [1]	1 (0.4%) [1]
Dehydration	1 (0.4%) [1]	0 (0.0%) [0]	1 (0.4%) [1]
Uterine leiomyoma	1 (0.4%) [1]	0 (0.0%) [0]	1 (0.4%) [1]
Psychotic disorder	1 (0.4%) [1]	0 (0.0%) [0]	1 (0.4%) [1]

Protocol Summary S7

**Study Title**	Efficacy and Safety of Fexinidazole in Patients with Stage-1 or Early Stage-2 Human African Trypanosomiasis (HAT) due to T.b. gambiense: a Prospective, Multicentre, Open-label, Cohort Study, Plug-in to the Pivotal Study
**Study Phase**	Cohort study
**Indication**	Human African Trypanosomiasis (HAT) due to *Trypanosoma brucei gambiense* at stage 1 or early stage 2
**Protocol Number**	DNDiHATFEX005 (V 5.0)
**Study Rationale**	HAT is a potentially fatal, neglected disease.<br>HAT is caused by a parasite that initially invades the blood, the lymph nodes and then the central nervous system. At the latter stage, the treatment of HAT requires adequate drug concentrations in the brain.<br><br>Patients with HAT are arbitrarily divided into two categories, stage 1 and stage 2, based on the fact that different drugs are used at each stage, i.e. pentamidine for stage 1 and nifurtimox-eflornithine combined therapy (NECT) for stage 2.<br><br>Dividing the patients into two categories is based on the differing capacity of the two drugs to cross the blood-brain barrier and reach the cerebrospinal fluid and brain (NECT crosses the blood-brain barrier while pentamidine does not), as well as on their toxicity and constraints related to their use, i.e. intramuscular for pentamidine and intravenous infusion for NECT. However, the distinction between stage-1 and stage-2 HAT is not always clear and simple. Indeed, some patients with so-called “early” stage-2 HAT can be treated with pentamidine, however the risk of relapse seems to increase with the number of white blood cells in the cerebrospinal fluid (1,2). Given the risk that patients who relapse will be lost to follow-up, most national HAT control programmes have elected to treat patients with “early” stage-2 HAT systematically with NECT.<br><br>In accordance with the WHO recommendations for clinical trials intended to demonstrate treatment efficacy in patients with stage-2 HAT (3), only patients with a white blood cell count > 20/µL in the cerebrospinal fluid and/or with trypanosomes in the cerebrospinal fluid are eligible to participate in the DNDiFEX004 study. These criteria were used in the pivotal study, DNDiFEX004 (4).<br><br>The present study will be a plug-in to the pivotal study DNDiFEX004 (4), which means that it will be possible to compare the results from the two studies since patients will be recruited simultaneously i.e. same centres and investigators, genetically homogeneous population.<br><br>As of 21 November 2013, 188 patients, i.e. around 125 patients receiving fexinidazole, had been randomised in the DNDiFEX004 study. No safety issues were identified on blinded data review. A total of 11 serious adverse events were reported, 3 of which were considered as possibly related to the investigational product and which resolved without sequelae. Overall, the safety profile was similar to that observed in earlier studies in healthy volunteers, and there were no treatment discontinuations. A meeting of the Data and Safety Monitoring Board was held. No safety issues were identified, and it was recommended to continue the study with the same design. No parasites were found in any patients at the End of Treatment visit, based on blinded review, and no relapses were observed among 80 patients who attended their 6-month follow-up visit, with the

	exception of one patient whose health status had been poor at inclusion and who died shortly after leaving hospital (blind not lifted).<br><br>In the DNDiFEX004 study, fexinidazole is administered by the oral route once daily after a meal: three 600-mg tablets daily for 4 days, followed by two 600-mg tablets daily for the next 6 days.<br><br>Pharmacokinetic analysis of the first 39 patients treated with fexinidazole in the DNDiFEX004 study showed that mean exposure to the M2 metabolite, the more active metabolite, in the cerebrospinal fluid was 2.6 times higher than the minimum inhibitory concentration used as the target value based on animal data collected in preclinical efficacy studies. Blood M1 and M2 concentrations were higher in patients than in healthy volunteers. The cerebrospinal fluid-to-blood ratio was around 31% for M2 and 52% for M1.
**Study Objectives**	Primary Objective<br><br>• To demonstrate that the success rate of treatment with fexinidazole at 1-year follow-up in patients with stage-1 or early stage-2 HAT is greater than 80%. An 80% success rate is considered to be inadequate and unacceptable.<br><br>Secondary Objectives<br><br>• To verify whether the success rate of treatment with fexinidazole varies depending on the stage of the disease, i.e. stage 1 or early stage 2, and, if the difference between the stages is significant, to show that the success rate is greater than 80% and consistent with the historical success rate reported with NECT in patients with early stage-2 HAT and with pentamidine in patients with stage-1 HAT.<br>• To verify whether the success rate of treatment with fexinidazole varies depending on the WBC count in the CSF prior to treatment initiation.<br>• To study changes in the success rate over time.<br>• To assess the safety of fexinidazole and verify whether the safety profile of fexinidazole is similar to the historical safety profile reported with pentamidine.
**Primary Endpoint**	**Efficacy**<br><br>The outcome, i.e. success or failure, observed at the test-of-cure visit, 12 months after the end of treatment.
**Secondary Endpoints**	**Efficacy**<br><br>The outcome, i.e. success or failure, observed at each visit between the end of treatment and 18 months.

	**<u>Safety</u>**<br>• Occurrence of adverse events at grade ≥ 3, including laboratory abnormalities, during the observation period. Adverse events will be graded according to the Common Toxicity Criteria for Adverse Events of the National Cancer Institute, Version 4.03.<br>• Occurrence of any adverse events, at any grade, during the observation period.<br>• Occurrence of any treatment-related adverse events (grade ≥ 3 and any grade) during the observation period.<br>• Occurrence of any serious adverse events between the first intake of the investigational product and the end of the follow-up period (18 months).
**Study Design**	This is a multicentre, open-label, cohort study with a single group of patients, stratified by self-weighting into 2 strata: patients with stage-1 HAT and patients with early stage-2 HAT.<br>At least 202 patients in at least 8 centres will participate in the study. The study is a plug-in to the pivotal study DNDiFEX004.
**Inclusion and Exclusion Criteria**	**Inclusion Criteria**<br>▪ Signed informed consent form.<br>▪ 15 years of age or older.<br>▪ Male or female.<br>▪ Able to ingest at least one complete meal per day (or at least one sachet of Plumpy'Nut®).<br>▪ Karnofsky score > 50.<br>▪ Evidence of trypanosomes in blood or lymph.<br>▪ No evidence of trypanosomes in CSF.<br>▪ Having a permanent address and able to comply with the schedule of follow-up visits.<br>▪ Willing to be hospitalised to receive treatment.<br><br>**Exclusion Criteria**<br>▪ Severe malnutrition, defined as Body Mass Index < 16.<br>▪ Unable to take medication by the oral route.<br>▪ Pregnancy or breast-feeding (for women of child-bearing potential, a urine pregnancy test will be performed within 24 hours prior to the start of treatment).<br>▪ Clinically significant medical condition (other than HAT) that could, in the opinion of the Investigator, jeopardise the patient's safety or interfere with participation in the study, including, but not limited to significant liver or cardiovascular disease, suspected or proven active infection (including HIV infection), CNS trauma or seizure disorder, coma or consciousness disturbances.<br>▪ Severely deteriorated general status, including as a result of cardiovascular shock, respiratory distress or end-stage disease.

	▪ Any condition (excluding HAT-specific symptoms) that affects the patient's ability to communicate with the Investigator as required to complete the study.<br>▪ Any contraindication to imidazole drugs, i.e. known hypersensitivity to imidazoles.<br>▪ Prior treatment for HAT in the previous 2 years.<br>▪ Prior enrolment in the study or prior intake of fexinidazole.<br>▪ Foreseeable difficulty complying with follow-up, including migrant worker, refugee status, itinerant trader.<br>▪ Active alcohol or drug addiction.<br>▪ Clinically significant laboratory test abnormality, including for example:<br>o alanine aminotransferase and/or aspartate aminotransferase more than 2 times the upper limit of normal (ULN),<br>o total bilirubin more than 1.5 x ULN,<br>o severe leukopenia at < 2000/mm^3,<br>o potassium < 3.5 mmol/L,<br>o any other clinically significant laboratory test abnormality (see Investigator manual for details).<br>▪ Pregnancy confirmed by a positive urine pregnancy test within 24 hours prior to the start of treatment.<br>▪ QTcF interval ≥ 450 msec on automatic reading, if the first reading is abnormal, a second reading will be performed at least 10 to 20 min later after placing the patient in the resting position.<br>▪ Not tested for malaria and/or not having received appropriate treatment for malaria.<br>▪ Not having received appropriate treatment for soil-transmitted helminthiasis.<br><br>For the purposes of the study, patients will be stratified according to the following criteria:<br>▪ Patients at stage 1 : CSF WBC ≤ 5/µL<br>▪ Patients at early stage 2: CSF WBC 6 to 20/µL<br>For the purposes of the study, the strata will be self-weighted. It is planned to include at least 101 patients par stratum.
**Study Duration**	Each patient's participation will last approximately 19 months and will include:<br>• pre-treatment period,<br>• treatment period of 10 days (D1 to D10),<br>• hospitalisation for 1 to 7 days after treatment,<br>• additional follow-up visit at 9 weeks after D1, i.e. between D64 and D70),<br>• out-patient follow-up with visits at 6, 12 and 18 months.

**Investigational Product**	**<u>Investigational Product</u>**<br>Fexinidazole, 600-mg tablets to be taken by the oral route after the main meal:<br>• 1800 mg (3 tablets) in one daily intake for 4 days,<br>• and then 1200 mg (2 tablets) in one daily intake for 6 days.<br>The total duration of treatment will be 10 days.
**Statistical Analyses**	**<u>Analysis Sets</u>**<br>The primary analysis will be performed on:<br>• the intent-to-treat population, comprising all patients who received at least one dose of fexinidazole;<br>• the per-protocol population, comprising all patients who adhered to the protocol with no major violations that could interfere with the efficacy analysis.<br>Sensitivity analyses will be performed on the following populations: treatment completers, evaluable patients, i.e. those with a known CSF WBC count at 12 months of follow-up or later and the per-protocol population.<br>**<u>Analyse</u>**<br>The primary analysis will be performed on the intent-to-treat population and will be based on the outcome, i.e. success or failure, of treatment with fexinidazole at 12 months of follow-up. If the lower limit of the 95% confidence interval is ≤ 80% then the study is a failure, otherwise it is a success.<br>The 80% limit was set by substracting the 13% margin (see DNDiFEX004) to the expected global success rate with the reference treatment currently used. By combining the historical success rate for Pentamidine (stage 1) to the success rate for NECT (stage 2), a global success rate of 93% at 12 months in a hypothetical group treated with the appropriate reference treatment can be expected.<br>The secondary analyses will assess whether the success rate varies depending on the stage of HAT, using a Fisher exact test to compare the rates in the two strata. The correlation between the success rate and the baseline CSF WBC will be investigated using a logistic regression. Finally, the time-course of the failure rate will be studied using the Kaplan-Meier method.<br>**<u>Sample Size</u>**<br>The sample size had initially been set at a minimum of 101 patients per stratum, i.e. at least 202 patients in total, with a maximum of 300 patients. However, the prevalence of stage-1 HAT is in fact seven times higher than the prevalence of early stage-2 HAT (N = 135 after 11 months of enrolment). It therefore seems unlikely that the initial objective of enrolling at least 101 patients at early stage-2 will be reached. Consequently, the objective of enrolling 101 patients will only be reached for stage 1.
**References**	1. Doua F, Miezan TW, Sanon Singaro JR, Boa Yapo F, Baltz T. The efficacy of pentamidine in the treatment of early-late stage Trypanosoma brucei gambiense trypanosomiasis. *Am J Trop Med Hyg* 1996;**55**:586-8.<br>2. Lejon V, Legros D, Savignoni A, Etchegorry MG, Mbulamberi D, Büscher P *Journal of neuroimmunology* 2003;**144**;132-8.<br>3. Recommendations of the Informal Consultation on Issues for Clinical product Development for Human African Trypanosomiasis, Geneva, 9-10 September 2004

4. Mesu V, Kalonji WM, Bardonneau C et al. Oral fexinidazole for late-stage African Trypanosoma brucei gambiense trypanosomiasis: a pivotal multicentre, randomised, non-inferiority trial. *Lancet* 2018; **391**:144-54.

Schedule of Study Procedures S8

**Protocol-planned procedures and forms to be completed**	**Pre-screening and Screening**	**Baseline**	**Treatment period**										**End-of-Treatment Visit until End-of-Hospitalisation Visit**		**Follow-up period (months)**
Timepoint →	D-15 to D-1	*D-4 to D-1*	*D1*	*D2*	*D3*	*D4*	*D5*	*D6*	*D7*	*D8*	*D9*	*D10*	*D11* *(EOT)*	*D11-18* *(EOH)*	6 – 12 – 18 months
Detection of parasite in blood and/or lymph	x												x		x
Lumbar puncture (parasite and white blood cells in CSF)	x														x
Informed consent (**before any additional medicines or study-specific procedures**)	**x**	**Check**													
Pretreatment of helminthiasis (+ 3-day recovery period)	x														
Rapid diagnostic test and/or thick blood smear for malaria	x														
Pretreatment of malaria if necessary (+ 3-day recovery period)	x														
Karnofsky score	x	x											x		x
Urine pregnancy test		X**												x	
Inclusion and exclusion criteria	x	x													
Demographic data	x														
Medical history	x														X [1]
Signs and symptoms of HAT		x												x	x
Vital signs	x	x					x			x			x	(x)*	x
Physical and neurological examination		x					x			x			x	(x)*	x
Haematology and biochemistry §		X					X						X		X[4]
Urine analysis §		X											X		
Safety ECG §		X											X		
Administration of fexinidazole			X	X	X	X	X	X	X	X	X	X			
Adverse event (AE)[2] collection			X	X	X	X	X	X	X	X	X	X	X	(x)*	
Serious adverse event (SAE) collection from signature of consent form to last study visit		X	X	X	X	X	X	X	X	X	X	X	X	(x)*	X
Collection of concomitant medication	X[3]	X	X	X	X	X	X	X	X	X	X	X	X	(x)*	

** Assessments to be performed only if EOH visit is different from EOT visit.*

[§] *Repeat tests possible, if needed, i.e. if result was abnormal on previous assessment.*
*** Pregnancy test to be performed on D-1 (i.e. within 24 hours prior to the start of fexinidazole.*

[1] *Record new events since previous visit.*

[2] *In addition, any adverse event that occurs after the AE reporting period and considered as possibly treatment-related by the Investigator must be reported.*

[3] *Including prior medication.*

[4] *At 9 weeks after D1, only sampling for haematology and biochemistry, physical examination (including vital signs) and neurological assessment are to be performed.*

[5] *At 6M visit, sampling for haematology and biochemistry*
